# An empirical method to cluster objective nebulizer adherence data among adults with cystic fibrosis

**DOI:** 10.2147/PPA.S131497

**Published:** 2017-03-24

**Authors:** Zhe H Hoo, Michael J Campbell, Rachael Curley, Martin J Wildman

**Affiliations:** 1 School of Health and Related Research (ScHARR), University of Sheffield; 2 Sheffield Adult Cystic Fibrosis Centre, Northern General Hospital, Sheffield, UK

**Keywords:** cystic fibrosis, medication adherence, nebulizers and vaporizers, epidemiologic methods, cluster analysis

## Abstract

**Background:**

The purpose of using preventative inhaled treatments in cystic fibrosis is to improve health outcomes. Therefore, understanding the relationship between adherence to treatment and health outcome is crucial. Temporal variability, as well as absolute magnitude of adherence affects health outcomes, and there is likely to be a threshold effect in the relationship between adherence and outcomes. We therefore propose a pragmatic algorithm-based clustering method of objective nebulizer adherence data to better understand this relationship, and potentially, to guide clinical decisions.

**Methods to cluster adherence data:**

This clustering method consists of three related steps. The first step is to split adherence data for the previous 12 months into four 3-monthly sections. The second step is to calculate mean adherence for each section and to score the section based on mean adherence. The third step is to aggregate the individual scores to determine the final cluster (“cluster 1” = very low adherence; “cluster 2” = low adherence; “cluster 3” = moderate adherence; “cluster 4” = high adherence), and taking into account adherence trend as represented by sequential individual scores. The individual scores should be displayed along with the final cluster for clinicians to fully understand the adherence data.

**Three illustrative cases:**

We present three cases to illustrate the use of the proposed clustering method.

**Conclusion:**

This pragmatic clustering method can deal with adherence data of variable duration (ie, can be used even if 12 months’ worth of data are unavailable) and can cluster adherence data in real time. Empirical support for some of the clustering parameters is not yet available, but the suggested classifications provide a structure to investigate parameters in future prospective datasets in which there are accurate measurements of nebulizer adherence and health outcomes.

## Introduction

Cystic fibrosis (CF) is a multisystem condition due to a genetic defect resulting in dysfunctional cystic fibrosis transmembrane conductance regulator (CFTR) protein.^1^ The lungs are the main organ affected – people with CF are vulnerable to recurrent infection, which leads to progressive lung damage and respiratory failure.^1^ Median survival has improved from ~6 months when CF was first recognized in 1938 to around 45–50 years due to improved treatment options and quality of care,^2^,^3^ but ~80% of all mortality in CF is still due to respiratory failure.^4^ An important maintenance treatment in CF is therefore inhaled therapies typically consisting of nebulized antibiotics and mucolytics, which have been proven to be efficacious in maintaining lung health.^5^,^6^ We have described this as “efficacious” rather than “effective”, since adherence in randomized controlled trials is 80%–100%,^7^ whereas real world data among adults with CF suggest that adherence is less than 50%.^8^,^9^ Such low levels of adherence limit the benefits derived from the new inhaled therapies that have been introduced over the past 20 years, despite the efforts of clinicians who are prescribing more of these to people with CF.^10^,^11^

The importance of increasing adherence to inhaled therapies is widely recognized among the CF community.^12^ An important step in developing strategies to improve adherence is the accurate quantification of adherence level, so that change can be measured and understood.^13^ In CF, the technology to accurately capture date- and time-stamped objective adherence data with tamper-proof chipped nebulizers is available for clinical use.^14^,^15^ However, data still need to be analyzed with the appropriate methods to quantify adherence accurately. We have recently published a methodology paper to explain the methods of calculating “normative adherence”, which better reflects treatment effectiveness compared to unadjusted adherence, by taking into account characteristics of a person with CF when defining the minimum required treatment regime.^16^ In this paper, we explain the rationale for clustering adherence data and present a pragmatic, algorithm-based method for clustering objective adherence data downloaded from chipped nebulizers.

We acknowledge that clustering will typically involve judgment, and different judgments may create different clusters. We have made judgments driven by a pragmatic approach to data that allow data to be clustered readily in routine practice with the aim of generating a systematic clustering strategy, that then can be subjected to empirical testing in prospective data and a paper doing this will follow.

### The purpose of clustering adherence data

Nebulizer adherence is a behavior, and the study of behavior has traditionally involved either group-level comparison (nomothetic approach) or analysis of individual-level longitudinal data (idiographic approach).^17^ Each approach has advantages and disadvantages. The nomothetic approach allows us to understand average behavior across the population.^18^ However, the average group result may conceal important granular details crucial to the understanding of the behavior of individuals,^18^ hence the increasing interest in the use of idiographic approach within health psychology.^19^ Although everyone may have unique behavioral patterns, clustering offers a compromise between the nomothetic and idiographic approaches, by identifying individuals with patterns of behavior that can be mapped within homogenous subgroups (clusters) that can accommodate a number of individuals with patterns that are similar enough to allow meaningful groupings.^18^ This allows homogenous subgroups to be studied, which may identify generalizable trends with a better understanding of how that relates to the group and to individuals.

Clustering of objective nebulizer adherence would involve the categorization of continuous data. This may be perceived as wasting valuable information and reducing statistical power for comparison.^20^ However, it has been argued that loss of information is small if there is an adequate number of categories to represent the continuous variable.^20^ A common reason for categorizing a continuous variable is to study the association between variables that are not linearly related.^21^ The fundamental purpose for supporting nebulizer adherence among adults with CF is to improve health outcomes, yet there is most likely a threshold effect for nebulizer adherence in relation to health outcomes. Increasing adherence from 1% to 5% would be a 5-fold increase, yet such a low level of adherence is not associated with good health outcomes.^9^,^22^ Similarly, a 5-fold increase in adherence from 90% to 450% is unlikely to give additional benefits. However, a 5-fold increase in adherence from 20% to 100% is very likely to have clinical benefits by reducing the frequency of pulmonary exacerbations and decreasing healthcare costs.^9^,^22^ Given the likely threshold effect of nebulizer adherence, clustering adherence data makes sense when we are analyzing data, to better understand the relationship between adherence levels and the expected health benefits. Indeed, it is anticipated that clustering of adherence data will allow more meaningful levels of adherence targets to be set by helping us to understand how much treatment is enough. In addition, adherence clusters might well allow clinicians to make a better judgment of the cause for deterioration in lung health based on objective nebulizer adherence data.

**Figure 1:**
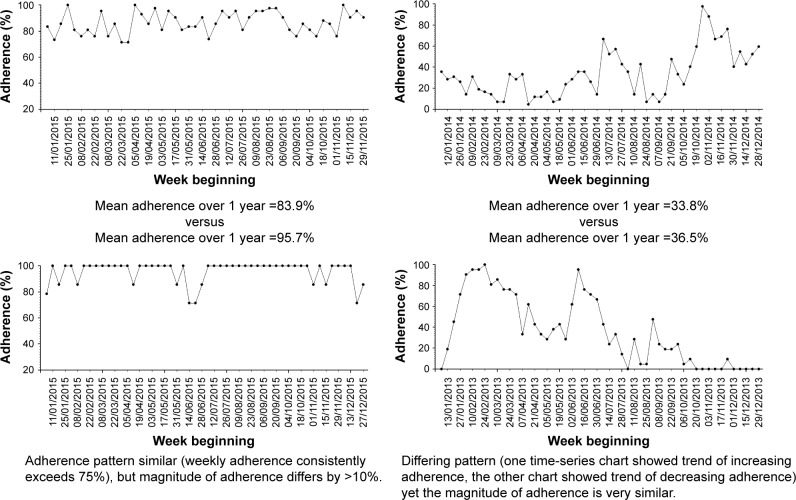
Examples of time-series adherence charts to highlight the importance of considering both the magnitude and the variability of adherence.

Previous studies have demonstrated that health benefits of medication adherence are likely to depend on both the magnitude and the variability of adherence.^23^,^24^ Yet, adherence levels are typically quantified in terms of the magnitude only, partly because some methods of capturing adherence data, eg, pharmacy refill data, could not identify variation in medication use.^25^ The availability of tamper-proof chipped nebulizers in CF means that detailed date- and timestamped adherence data are available to study the pattern of adherence.^26^ We provide examples of different time-series adherence patterns in Figure 1 to highlight the importance of quantifying both the magnitude and the variability in adherence. By identifying different clusters of adherence pattern taking into account both the magnitude and variability, there is potential for determining the level of adherence that is needed to improve health outcomes, or to potentially target appropriate adherence interventions.

### Criteria for the methods used to cluster adherence data

Several methods are available for clustering time-series adherence data,^27^ and the purpose of clustering should be taken into account when selecting the appropriate clustering method. Given that the magnitude and variability of adherence may influence health outcomes, it will be important that both magnitude and variability are taken into account in the clustering method.

**Figure 2:**
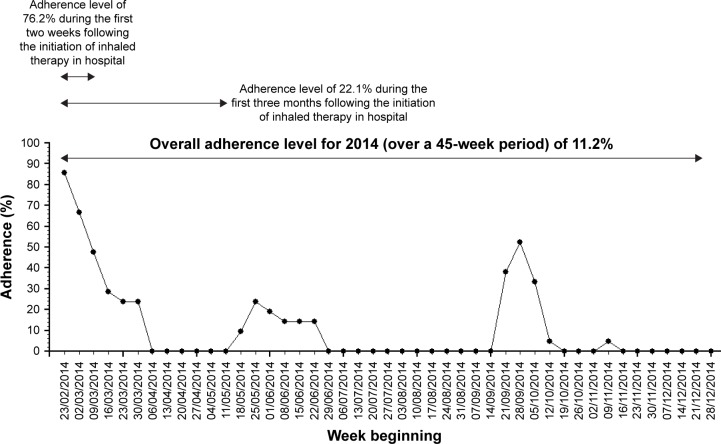
An example of the impact of using different data duration to infer the annual adherence level.

For a clustering method to help guide day-to-day clinical management of people with CF (eg, by setting an adherence target or in understanding the likely contribution of nebulizer adherence to lung function decline in someone reviewed in a CF clinic), an important practical consideration is the ability to cluster adherence data in real time. Another equally important consideration is to avoid a clustering method where the addition of data from new subjects could alter the cluster of previously clustered subjects. Finally, the clustering method will be most useful if it can be applied for all available adherence data. For example, if a clustering method required 12 months of data – not everyone will have complete annual adherence data – some may be started on inhaled treatment in the middle of the year, and missing data may occasionally occur, eg, due to machine malfunction or saturation of electronic data capture capacity. Data imputation to infer complete annual adherence is difficult since data are not missing at random.^28^ Since adherence in long-term conditions will tend to decrease with time,^29^,^30^ when only a short duration of data are available there is a risk of overestimating the long-term adherence rate. In Figure 2, we illustrate an example of high adherence level following initiation of inhaled therapy that then declined, demonstrating the potential for error if short runs of adherence data are used to infer overall steady state or customary adherence. We have chosen to use a minimum period of 3 months to ensure that the adherence classification takes into account a period of time that is long enough to reflect a meaningful period of adherence behavior likely to have a reasonable probability of having some relationship with lung health.

### Processing time-series adherence data for clustering

All clustering methods require a clustering technique/algorithm and the relevant variables from a dataset for clustering. For time-series data, there are three broad approaches to “process” the variables for clustering: use the actual time-series pattern (ie, raw data-based), use summary measures derived from the time-series data (ie, feature-based), and use model parameters from time-series data or identify different time-series model (ie, model-based).^31^

### Existing techniques to cluster “processed” time-series adherence data

Most clustering techniques can be applied for any of the three approaches for processing time-series data. Several techniques for clustering data exist.

An example of a data-driven clustering technique is the “principal components analysis” (PCA), which uses orthogonal transformations to combine a group of correlated variables into linear functions of these which are uncorrelated.^32^ PCA has also been applied to time-series adherence data to identify different adherence clusters^33^ and can work with either of the three different variable approaches. An argument to support the use of data-driven clustering methods is the lack of need for a priori assumptions which may introduce bias. However, it is important to note that assumptions are still needed in terms of deciding which variables are clinically important enough to be analyzed with the data-driven clustering method. Indeed, three different studies aiming to identify phenotypes of COPD using different clinical variables have yielded different groups of clusters.^34^ It should be noted that the generation of different clusters is not necessarily a problem if the clusters’ validity in terms of predicting outcomes can be empirically tested in prospective datasets. Hence the main issue is identifying a rational and reproducible approach to generate clusters that can be used in clinical practice, and is then suitable for empirical confirmation in prospective data-sets. Data-driven methods also tend to require complete data for cluster analysis (eg, only 92 of the 124 eligible participants in the study by Yeo et al were analyzed due to missing data),^33^ which is a disadvantage when nebulizer adherence datasets are of varying duration and imputation is difficult due to the missing not at random nature of adherence data. Perhaps the biggest disadvantage of a data-driven clustering technique is with clustering data from new subjects in real time. Regularly repeating the clustering process with the introduction of any data from new subjects is not an option, since that could assign a previously clustered dataset to new clusters. Therefore, the only practical way to use data-driven clustering methods in a clinical context is to cluster a representative sample of adherence dataset to generate rules to define newly acquired adherence data into existing clusters. However, rules based on time-series pattern (or even time-series summary measures) may not be straightforward to apply, so this would introduce subjectivity in defining the cluster for new adherence data and also potentially result in difficulty of dealing with new adherence data that were deemed “unclassifiable” within the existing rules.

There are also other data-driven clustering techniques designed specifically to deal with time-series adherence data. An example is the Typology of Temporal Patterns approach, which uses dynamic cluster analysis to identify different adherence clusters based on the raw treatment adherence time-series pattern.^18^ However, the methods also have similar disadvantages to using PCA to cluster time-series adherence data, ie, the need for equal adherence data duration, and it is difficult to perform the clustering in real time.

**Figure 3:**
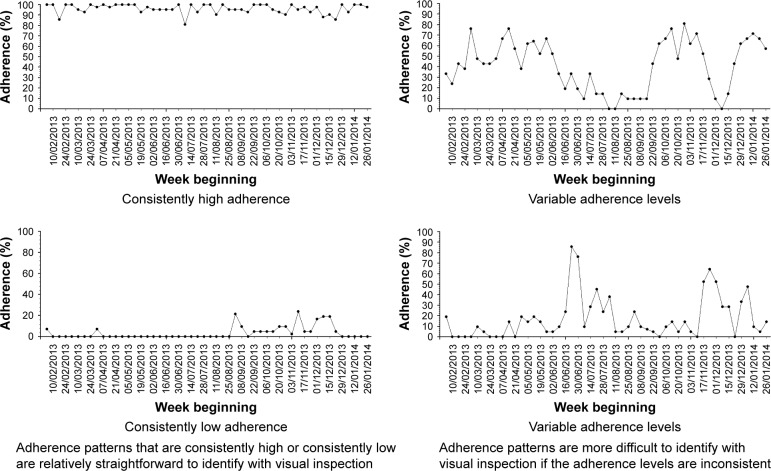
Examples of adherence patterns that are relatively easy and those that are more difficult to identify with visual inspection.

An alternative clustering technique for time-series data is using visual inspection to identify different adherence patterns.^35^ Visual inspection could be criticized as being subjective, but is used in practice, eg, almost all radiology images are read and interpreted by clinicians based on pattern recognition from visual inspection.^36^ This is nonetheless a labor-intensive process that also requires significant investment in staff training in order to reliably cluster adherence data with visual inspection. Whilst some adherence patterns are relatively easy to identify with visual inspection, there are other more ambiguous patterns whereby visual inspection is less useful (Figure 3).

### A brief description of our proposed adherence data clustering method

Given the limitations of the current available techniques to cluster objective nebulizer adherence data, we propose an algorithm-based clustering method which is able to handle adherence data of varying duration (including dataset with missing data) without the need for imputation. The proposed method also allows for real-time clustering of new adherence data, taking into account both the magnitude and variation in adherence. The clustering method consists of three separate steps, which we describe in the next section. We also provide exemplar cases to illustrate how the proposed clustering method can be applied.

As outlined previously, there is no perfect way to cluster adherence data in a clinical context, however, the approach we have adopted allows us to use reproducible methods to generate adherence clusters that can then be explored empirically in prospective datasets in terms of the relationship between adherence and health outcomes (eg, frequency of pulmonary exacerbations).

## Using an algorithm-based method to cluster objective nebulizer adherence data

**Figure 4:**
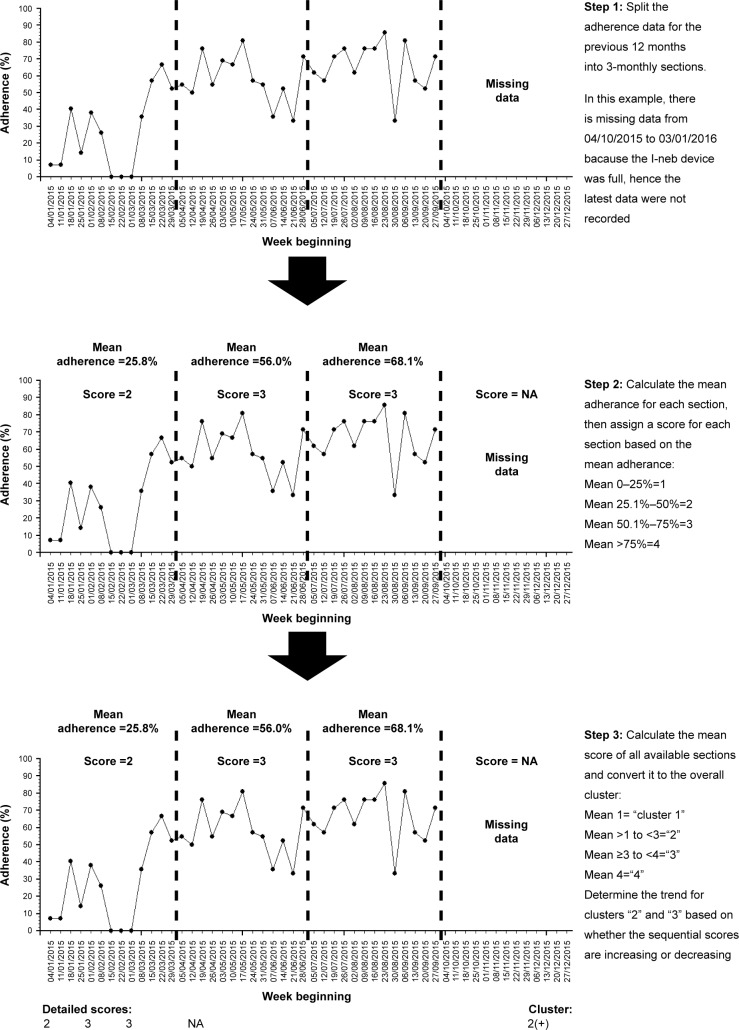
Summary of the steps involved in clustering adherence data using our proposed algorithm-based technique. **Notes:** It is important to display both the detailed scores for each section and the overall cluster for the data, so that the overall cluster can be interpreted accurately. In this example, the overall adherence is low, but the adherence is improving with time over a 9-month period from January to September 2015.

We describe each step involved in clustering adherence data using our proposed algorithm-based method and summarize the steps involved in Figure 4. The time-series charts displayed in Figure 4 are for illustrative purpose only – adherence data can be clustered with the described method by calculating normative adherence for 3-monthly sections without plotting time-series charts.

### Step 1: splitting the previous 12 months of adherence data into 3-monthly sections

Splitting annual adherence data into four 3-monthly sections is an empirical approach that balances the tension between detecting variation in adherence over time, whilst sampling enough sustained behavior to create an expectation that the behavior might be related to other downstream effects such as lung health. It also allows people who have less data to be clustered using the same technique with a minimum data capture period of only 12 weeks required to classify people’s behavior.

The 3-monthly period represents a good compromise between detail and practicality. Shorter periods of adherence (eg, monthly) preserves more information but as demonstrated in Figure 2, short runs of adherence data may poorly reflect the underlying adherence patterns that might be expected to relate to more distal outcomes such as lung health. For example, short time periods may be prone to overestimating underlying adherence if particularly good periods of adherence, eg, when people are hospitalized, comprise most of the relatively short segment of data analyzed. On the other hand, splitting the adherence data into longer periods (eg, 6 months) would produce insufficient numbers of sections to understand the variation of adherence over a 1-year period and also mean ignoring a subgroup of people with only 3–6 months’ worth of adherence data in any given 1-year period.

### Step 2: assigning each 3-month section with a score for the mean adherence level of that section

The mean normative adherence^16^ for each 3-monthly section should be calculated. This allows each section to be scored based on mean adherence, with mean adherence of 0 to 25% assigned a score of “1”, mean adherence of 25.1% to 50% assigned a score of “2”, mean adherence of 50.1% to 75% assigned a score of “3”, and mean adherence of 75.1% or above assigned a score of “4”.

### Step 3: aggregating the scores of each 3-monthly section to determine the final cluster and displaying the results

People with 12 months of adherence data over the previous year will thus have four sections, while people with 3 months of adherence data will have one section. The mean score for all the sections in the previous year is grouped to determine the adherence cluster. A mean score of 1 defines cluster “1”. A mean score of >1 but <3 defines cluster “2”. A mean score of ≥3 to <4 defines cluster “3”. A mean of score of 4 defines cluster “4”. In effect, all the mean scores are rounded down to the nearest whole number to determine the overall cluster, except for scores between 1.1 and 1.9 that were rounded up to “2”. The purpose of aggregating the scores in this manner is to separate the group with consistently high adherence (cluster “4” can only be achieved if all sections are scored “4”) and the group with consistently low adherence (cluster “1” can only be achieved if all sections are scored “1”). This is consistent with the groups that are easily identifiable with visual inspection of time-series pattern as described in Figure 3.

We recognize that those with moderate adherence (clusters “2” and “3”) may well display a trend of increasing or decreasing adherence with time. The trend is relevant for clinicians when monitoring the adherence of people with CF. For example, people with declining adherence require diagnosis for the cause of adherence decline and the necessary intervention, whereas people with a trend of improving adherence may only require regular adherence feedback and encouragement. The trend can be understood if all the individual scores for each section are displayed along with the overall cluster score. For example, someone with sequential section scores of “1”, “2”, “2”, and “3” would have an overall cluster labeled “2”, and the trend of improving adherence is apparent. On the other hand, someone with sequential section scores of “4”, “4”, “2”, and “1” would also have an overall cluster labeled “2”, but a trend of declining adherence is present. For convenience, an improving trend can be denoted with a “+” sign after the cluster number and a declining trend can be denoted with “−” sign whilst the “o” sign can be used to denote the lack of any obvious trend. The person with sequential section scores of “1”, “2”, “2”, and “3” would therefore be in cluster “2(+)”, whilst the person with sequential section scores of “4”, “4”, “2”, and “1” would therefore be in cluster “2(−)”. By taking into account the possible adherence trends for clusters “2” and “3”, there are in effect eight different clusters – “1”, “2(−)”, “2(o)”, “2(+)”,“3(−)”, “3(o)”, “3(+)”, and “4”. However, in terms of analyzing the effect of adherence on health outcomes, we anticipate that only four different clusters (“1” to “4”) matter, since the number of exacerbations will probably even out over a 1-year period (those with declining adherence are more likely to have exacerbations during the latter part of the period, while those with improving adherence are more likely to have exacerbations during the earlier part of the period).

Another important reason for displaying all the individual section scores is to make clear the amount of adherence data that are available for the previous 12 months, so that clinicians can make a better judgment regarding the adherence status of a person with CF. For example, someone with sequential section scores of “4”, “4”, “4”, and “4” would be in the same cluster as someone with only 3 months of high adherence (ie, sequential section scores of “NA”, “NA”, “NA”, and “4”). A clinician interpreting the cluster scores can be confident that the first individual has stable and high adherence over a 12-month period, whereas the second individual only has 3 months’ worth of adherence data.

## Practical examples of clustering adherence data using the algorithm-based method

**Figure 5:**
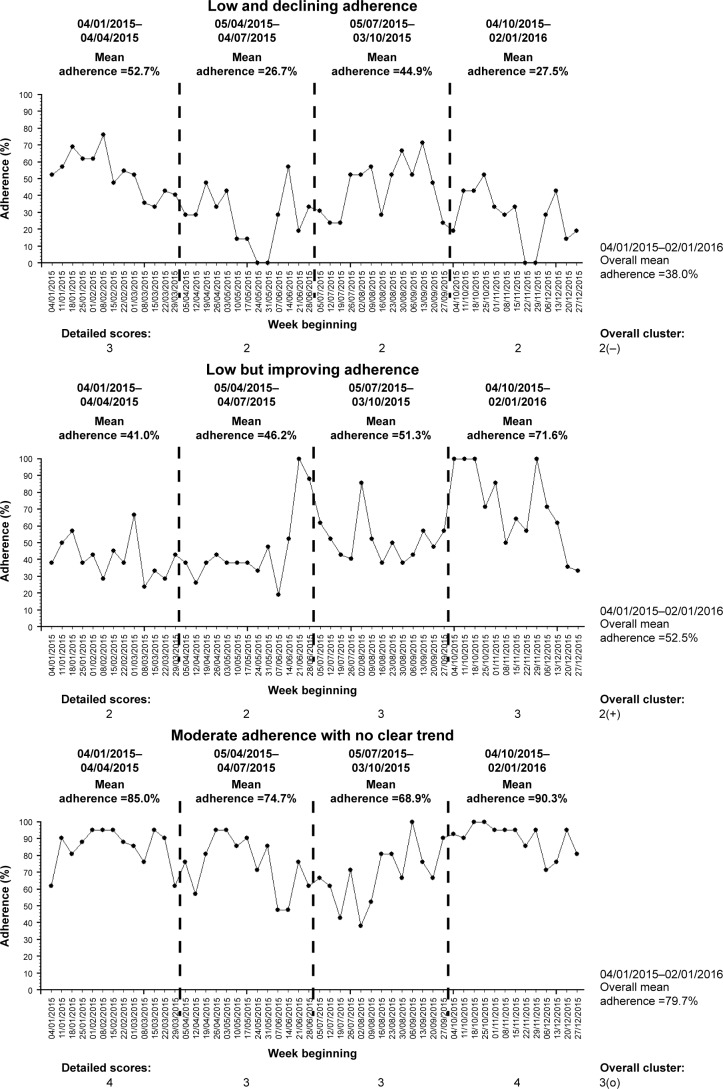
Weekly normative adherence time-series charts for the three example cases. **Note:** 3(o), no clear adherence trend.

We present three examples to illustrate the use of our proposed algorithm-based method to cluster adherence data. These examples are summarized in Table 1 and Figure 5. All the time-series charts displayed (Figure 5) are for illustrative purposes only and are not an essential component of the clustering process.

### Example A: low and declining adherence

Person A (Figure 5A) is in his early 40s and has chronic *Pseudomonas* in 2015 based on the Leeds definition.^37^ He has pancreatic insufficiency and CF-related diabetes. His best FEV_1_ in 2015 was 64% and he required 35 days of intravenous antibiotics throughout 2015. His nebulized treatments prescription consisted of once-daily dornase alfa and twice-daily colistimethate sodium alternating every 4 weeks with twice-daily tobramycin.

He has a complete 12 months’ worth of adherence data in 2015 from the week beginning 04/01/2015 to week beginning 27/12/2015, with an overall normative adherence of 38.0%. During the first 3 months of the period, his normative adherence was 52.7%, but his subsequent 3-monthly adherence levels were 26.7%, 44.9%, and 27.5%. He therefore scored “3” for his first 3-month section and “2” for the next three sections. This indicates a trend of decline in his adherence. His mean cluster score is 2.25, which equates to an overall cluster of “2”. Given the trend of decline, his overall detailed cluster is therefore “2(−)”.

Person A therefore has a low adherence with a trend of decline in his adherence for the 12-month period throughout 2015.

### Example B: low but improving adherence

Person B (Figure 5B) is in his mid-30s, has pancreatic insufficiency, and also has chronic *Pseudomonas* in 2015 based on the Leeds definition.^37^ His best FEV_1_ in 2015 was 78% and he required 33 days of intravenous antibiotics throughout 2015. He was on once-daily dornase alfa, along with twice-daily nebulized colistimethate sodium alternating every 2 weeks with twice-daily nebulized tobramycin.

He has a complete 12 months’ worth of adherence data in 2015 from the week beginning 04/01/2015 to week beginning 27/12/2015, with an overall normative adherence of 52.5%. During the first half of 2015, his 3-monthly normative adherence levels were 41.0% and 46.2%. His adherence levels continue to improve throughout 2015 to 51.3% during the third quarter and 71.6% during the final quarter of 2015. He therefore scored “2” for both 3-month sections during the first half of 2015 and “3” for both 3-month sections during the second half of 2015. This indicates a trend of improvement in his adherence. His mean cluster score is 2.5, which is rounded down to “2” for his overall cluster. Given the trend of improving adherence, his overall detailed cluster is therefore “2(+)”.

Person B therefore has an overall low level of adherence, but there is a trend of improving adherence during the 12-month period throughout 2015. Compared to Person A, Person B’s adherence has a different trend, which illustrates the ability of the proposed clustering method to account for both the variation and magnitude of objective nebulizer adherence.

### Example C: moderate adherence with no clear trend

Person C Figure 5C) is in her early 20s and has chronic *Pseudomonas* in 2015 based on the Leeds definition.^37^ She has pancreatic insufficiency and CF-related diabetes. Her best FEV_1_ in 2015 was 65% and she required 30 days of intravenous antibiotics throughout 2015. Throughout 2015, she was on once-daily dornase alfa and twice-daily nebulized colistimethate sodium.

She has a complete 12 months’ worth of adherence data in 2015 from the week beginning 04/01/2015 to week beginning 27/12/2015, with an overall normative adherence of 79.7%. During the first 3 months of the period, her normative adherence was 85.0%. Her 3-monthly adherence subsequently declined to 74.7% and 68.9%, before improving again to 90.3% during the last quarter of 2015. She therefore scored “4” for her first 3-month section, “3” for the next two sections, and “4” for the final section in 2015. This indicates no clear trend in her adherence over the 12-month period (initial decline followed by subsequent improvement). Her mean cluster score is 3.5, which is rounded down to “3” for her overall cluster. Given the lack of trend, her overall detailed cluster is therefore “3(o)”.

Person C therefore has a moderate adherence without a clear trend for the 12-month period throughout 2015. This example illustrates that it is possible to have relatively high overall nebulizer adherence of ~80%, yet there can be periods whereby adherence levels are relatively lower, hence the importance to understanding adherence over time.

## Discussion

We have described a pragmatic algorithm-based clustering technique that can be used in real time and can also cluster adherence data with varying data duration. The clustering technique consists of three related steps: splitting the data into 3-monthly sections, scoring each section based on mean adherence, and aggregating the scores to determine the overall cluster. This clustering technique recognizes the two distinct groups of adherence archetype – consistently low adherence (“cluster 1”, very low adherence) and consistently high adherence (“cluster 4”, high adherence). “Cluster 2” (low adherence) and “cluster 3” (moderate adherence) have more ambiguous patterns on visual inspection, and adherence data in those clusters can also display variation with time. “Cluster 2” and “cluster 3” are therefore separated into three different groups, each based on adherence trend (improving adherence, declining adherence, and no obvious trend).

Clustering of adherence data into the described categories, along with the “detailed” individual section scores, allows the adherence results and data completeness to be easily interpreted. In addition to potentially guiding the management of individual adults with CF, the categorization of adherence data also allows clinicians to gain a better understanding of the overall nebulizer adherence within their specialist CF centers. The usual summary measures for a continuous variable (eg, mean or median) do not readily inform clinicians regarding the distribution of the adherence levels within their center. For example, it is possible to achieve a mean adherence of 50% if everyone in that center has adherence of 50%, but mean adherence of 50% can also be achieved if 50% of the people in a particular center has mean adherence of 100%, while the other 50% has adherence of 0%. With the proposed clustering method, clinicians can identify the proportions of people in their center with “very low”, “low”, “moderate”, or “high adherence”. This also allows center comparisons using funnel plots to drive quality improvement initiatives,^38^,^39^ by comparing the proportion of high adherence for the different specialist CF centers.

The proposed algorithm-based clustering method can be automated; hence the clustering can be delivered within routine clinical practice via a software package. Objective adherence data captured from routinely available chipped nebulizers should be stored in a secure data repository, and the data in a repository can then be clustered using a software package. We proposed clustering nebulizer data as a starting point since objective data can already be captured within routine clinical practice.^14^,^15^ CF is a multisystem condition; hence the treatments are also necessarily multimodal, including physiotherapy for airway clearance, pancreatic enzyme replacement, nutritional supplementation, and management of CF-related diabetes.^1^ As technology advances, it is likely that most of the other CF treatments would also be chipped to routinely capture objective adherence data. For example, chipped airway clearance devices and chipped pill bottles (eg, Medication Event Monitoring System) have been used to objectively monitor adherence among people with CF in a research setting.^40^,^41^ The algorithm we described is versatile and can also be used to cluster adherence data from other devices.

The proposed clustering method does have some limitations. In studying variation over time, we proposed aggregating time-series adherence data into 3-monthly sections based on a balance between granularity and practicality. In addition, nebulized therapy in CF tends to have benefit that accumulates over time rather than instantaneously, and 3 months is a credible period over which effects might be apparent. Theory dictates that time-series data aggregated over longer periods (eg, over months) will lose more information compared to fine-grained (eg, weekly) analysis.^42^,^43^ There is thus uncertainty regarding the optimal period for aggregating adherence data to minimize information loss on data variation, and to avoid results being skewed by unpredictable short-term events, while keeping the clustering process simple enough. Similarly, there is also uncertainty regarding the optimal levels of scoring to assign to each 3-monthly section. We proposed an equal 4-level scoring system (from 1–4) because there is some evidence that adherence levels need to exceed 75% for good health outcomes.^9^,^22^ With our proposed system, mean adherence of 26% is scored the same as mean adherence of 50%. Increasing the number of scores (eg, using a 10-level scoring system instead) or changing the width of each score band could provide greater granularity and identify more subgroups,^20^ but at the cost of increased complexity. It is crucial to acknowledge the need to achieve a balance between “lumping” and “splitting” in cluster analysis. Identifying many different subgroups may not be that useful if the different subgroups behave similarly or respond similarly to different treatments.^44^ A pragmatic way to resolve the uncertainty regarding the aggregation period, level of scoring, and total number of adherence clusters that is clinically useful would be to test the variations of the proposed clustering method (eg, 2-monthly aggregation instead of 3-monthly aggregation, or scoring adherence over ten levels instead of four levels) in a well-defined prospectively collected adherence dataset linked to health outcomes. Indeed, the nebulizer adherence intervention randomized control trial funded by the National Institute of Health Research^45^ would provide a suitable dataset for fine-tuning the proposed clustering method.

In conclusion, we have proposed a pragmatic clustering method for objective nebulizer adherence data that could potentially improve the understanding of the relationship between adherence and health outcomes, allow center comparison with funnel plots, and also guide clinical decisions when monitoring adherence. Cluster analysis of adherence data in CF has been attempted using self-report data.^46^ The advantages of using objective adherence data for clustering include better accuracy and the ability to study adherence variability in detail.^8^,^26^ There is uncertainty regarding some of the parameters chosen for this proposed method of clustering, but these parameters can be fine-tuned using a well-defined adherence dataset linked to health outcomes.

## Figures and Tables

**Table 1:** Summary of the adherence clusters for the three example cases

Example	Detailed scores (mean adherence)				Overall cluster (mean adherence)	Interpretation
	First quarter of 2015	Second quarter of 2015	Third quarter of 2015	Fourth quarter of 2015		
Person A	3 (52.7%)	2 (26.7%)	2 (44.9%)	2 (27.5%)	2(−) (38.0%)	Has 12 months’ worth of adherence data throughout 2015. Overall adherence is low, and there is a trend of declining adherence
Person B	4 (85.0%)	3 (74.7%)	3 (68.9%)	4 (90.3%)	3(o) (79.7%)	Has 12 months’ worth of adherence data throughout 2015. Overall adherence is moderate. There is no clear adherence trend
Person C	2 (41.0%)	2 (46.2%)	3 (51.3%)	3 (71.6%)	2(+) (52.5%)	Has 12 months’ worth of adherence data throughout 2015. Overall adherence is low, but there is a trend of improving adherence
